# The Hospital Problem: Bogeys

**Published:** 1911-04-29

**Authors:** 


### The Hospital

A Journal of

The Medical Sciences and Hospital Administration.

.New Series. No. 221, Vol. IX. [No. 1293, Vol. L.] SATURDAY, APRIL 29, 1911

### The Hospital Problem: Bogeys.

Every process of reform, no matter how well considered, will have to meet the opposition raised by those who, through timidity or blind adherence to the obsolete, see in each evolutionary phase a sign of impending catastrophe and disaster. Nor are the suggestions for hospital reform in this country exempt from such conflict with prejudice on the one, and timorousness on the other hand. The unreasonable prepossessions demand a special criticism from which, for the moment, we refrain: the honest fear that by adopting some outlined course we may plunge ourselves into greater evils than those already besetting us needs less criticism than argument. Where such convictions are based on a dishonest wish to burke discussion by raising supposititious bogeys to frighten the cautious, no amount of argument will dispel them. It is probable that some at least of the opinions publicly expressed, in speech and in the Press, are built on such foundations, but the majority, fortunately, are buttressed not by obstinate and unreasoning presuppositions, but by a real solicitude for preserving what is good, even at the risk of at the same time maintaining that which is admittedly bad and amenable to reform, simply because the suggested emendations present for their thorough execution certain dangers which appear to be only too real.

One of these dangers, which has been very prominently referred to during the past few weeks, is the bogey of a despotic bureaucracy. It is alleged that by consolidating and concentrating the efforts, now divided and reft of much of their power through such division, of the voluntary hospitals, by bringing about a perfect system of co-operation between them and State institutions and organisations for the relief of sickness and distress, and by further arranging for a system of State insurance for invalidity and sickness—in short, by carrying out those measures of reform on which The Hospital has always insisted and which are now, we are happy to say, winning more and more public support as they are becoming better known and as their importance is becoming better realised—there will be created an immense charitable trust that will rival in malignity the much criticised American commercial trusts. Since the exposition of the methods favoured by certain of the more powerful combinations in this country and elsewhere, the public has come to attach what is perhaps an unjustifiably derogatory meaning to the word Trust, but that interpretation is essentially the one which attaches to the word when it is used as a bogey to frighten those who favour the extension of a copartnership scheme between the voluntary charitable institutions in this country. We must therefore accept it, however unjust and derogatory it may be. The want of logic that supposes such combinations must inevitably be inimical to the community because in certain cases they have proved to be immoral in a social as well as in an economic sense, scarcely needs comment. What asks for some replication is the argument that co-operation and concentration are bound to establish an "inimical" trust "such as is already apparent in the great funds." Apart altogether from the fact that this dictum cannot be supported by an itemised indictment: that the whole history of the King's Fund, the Saturday Fund, and (at least up to the present) of the Sunday Fund, amply refutes the accusation that these associations are offensively despotic; and that the very conditions under which they carry on their work prevent them from exercising an immoral influence on hospital policy, it is clear that those who have raised this Trust bogey do not realise the logical force of their own arguments. If a controlling body (controlling only in so far that it can recommend, and, if its recommendations are disregarded, show its dissatisfaction by withdrawing a portion of the institution's funds) which possesses undoubtedly a large moral and material influence—amassed, be it noted, by voluntary co-operation—and which represents not a self-interested group of individuals but the charitable public without distinction of class or degree—if such a body presents a danger to the community, how much more dangerous, then, must be the control of the hospitals by a department of State which is responsible only to one individual? The obvious reply to this presentation is, of course—Parliament. We are quite aware that there are many good people who are still firmly convinced that Parliamentary control of public departments is a reality. Such people lose sight of the fact that Parliamentary agitation has hardly ever succeeded in controlling or checking abuses in public charitable institutions to the same extent that the large funds have done. The reformatory scandals and the revelations made in Professor Moore's book and in Mr. Sydney Holland's letters regarding the amazing conditions prevalent in a Scottish infirmary are recent illustrations of the fact that parliamentary interference is slow, ineffective, and costly.

We need not unduly labour this point. What is clear is that the funds have never exercised their power in an immoral fashion. If they ever do so, the fault lies entirely with the public. There is here no question, there can be no question, of a Trust in the ordinary sense of the word. The directorate of these organisations is representative of the people who supply the funds. The claim that it should also be representative of the class that is benefited by these funds, a claim which has been lately raised, is open to argument, but the mere fact that this class is not represented does not make the directorate an arbitrarily despotic trust. The careful and unprejudiced consideration of the history of these great funds will convince anyone who takes the trouble to study statistics of finance, improvement, and extension, that their establishment has been of the utmost benefit to hospitals in this country. A central controlling board, responsible to the public, on which all sections of the community are represented, will not be a menace but a boon to institutional charity. Voluntaryism we have always defended, but it must be a controlled and directed exertion of voluntary effort, not, as now happens in some cases, a wasteful and divided display of voluntary energy which leads to overlapping, exhaustion, and stagnation.

